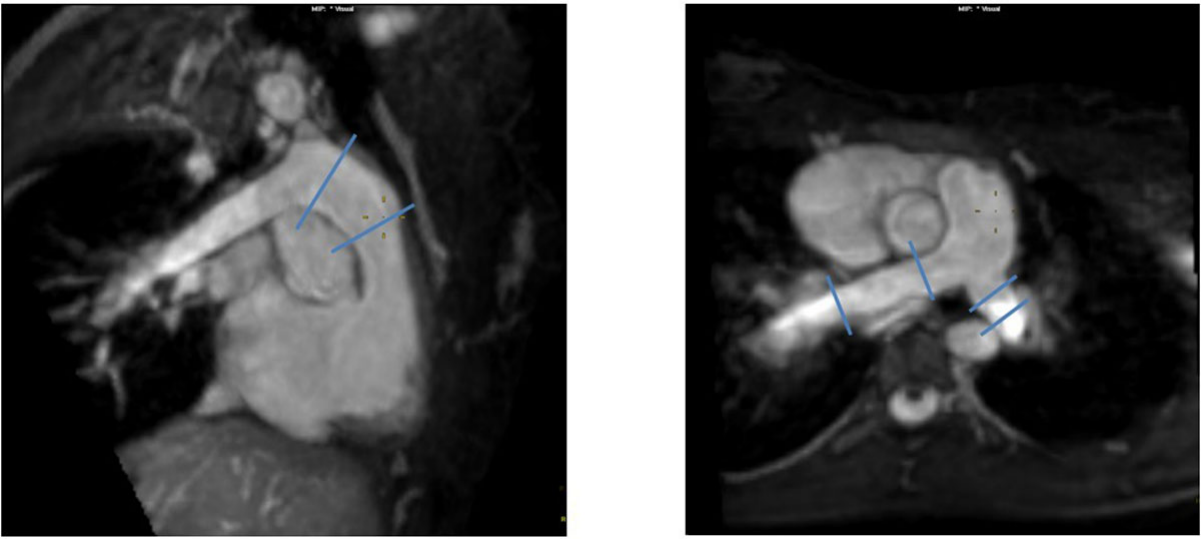# Comparison of great artery dimensions in 3-D dual-phase SSFP, compared with 3D CE-MRA and phase-contrast imaging (magnitude image)

**DOI:** 10.1186/1532-429X-17-S1-P44

**Published:** 2015-02-03

**Authors:** Aimin Sun, Srinivas Ananth Narayan, Gerald F Greil, Tarique Hussain, Kuberan Pushparajah, Aaron Bell, Sujeev Mathur, Reza Razavi

**Affiliations:** 1 Paediatric Cardiology, Evelina London Children's Hospital, Harrow, UK; 2 Diagnostic Imaging, Shanghai Children's Medical Centre, Shanghai, China

## Background

The dimensions of great vessels are measured in different methods in different institutes. The purpose of this study was to evaluate the benefits of 3D dual phase steady-state free-precession(3D-DP SSFP)for measuring great arteries dimension, compared with 3D contrast-enhanced magnetic resonance angiography (3D CE-MRA) and 2D phase contrast imaging (Magnitude image) (2DPC-MI), in order to find which was the most suitable and reproducible technique for follow-up.

## Methods

29 patients with repaired Tetralogy of Fallot or complete transposition of the great arteries after arterial switch operation (mean age 6.5yrs; range 6m to 25yrs) were included in the study. Cross-sectional diameter and area measurements were taken of the ascending aorta (Ao), main pulmonary (MPA) and branch pulmonary arteries (BPA) by using 3D DP SSFP, 3D CE-MRA and magnitude image of 2DPC-MI. Image quality was scored by a five-point scale (0 = invisible to 4 = excellent). Statistical comparison between 3D DP SSFP and other two techniques (2DPC-MI and 3D CE-MRA) was performed by using paired-t tests and Intraclass correlation coefficient.

## Results

All great artery cross-sectional measurements were significantly (P < 0.001) greater in systole than in diastole. Measurements (diameter and area) of great arteries were greatest for 2DPC-MI, followed by 3D SSFP in systole and 3D CE-MRA, and smallest for 3D DP SSFP in diastole. There was no significant difference of aortic measurements between 3D DP SSFP in systole and 3D CE-MRA, but significance was observed between 3D DP SSFP in systole and 2D PC-MI (P < 0.05). The measurements of MPA and BPA showed no significant difference for 3D DP SSFP in systole compared to other two techniques. Intra-observer agreement of aortic measurements was uniformly >0.95, with 2DPC-MI being the best, followed closely by 3D DP SSFP in systole, and 3D CE-MRA being the worst. The average image quality of 3D DP SSFP and 2DPC-MI were ≥3. But the image quality was significantly poorer for 3D CE-MRA compared to other two techniques (P < 0.001).

## Conclusions

All Ao and PA cross-sectional measurements were significantly (P < 0.001) greater in systole than in diastole. Measurements of Ao and PA were greatest for 2DPC-MI, followed by 3D SSFP in systole and 3D CE-MRA, and smallest for 3D DP SSFP in diastole. There was no significant difference of aortic measurements between 3D DP SSFP in systole and 3D CE-MRA, but significance was observed between 3D DP SSFP in systole and 2D PC-MI (P < 0.05). The measurements of MPA and BPAs showed no significant difference for 3D DP SSFP in systole compared to other two techniques. Intra-observer agreement of Ao measurements was uniformly >0.95, with 2D PC-MI being the best, followed closely by 3D DP SSFP in systole, and 3D CE-MRA being the worst. The image quality of 3D DP SSFP and 2D PC-MI scored≥3. But the image quality was significantly poorer for 3D CE-MRA compared to other two techniques (P < 0.001).

## Funding

The first author recieved an educational grant from Philips Healthcare.

**Figure 1:**
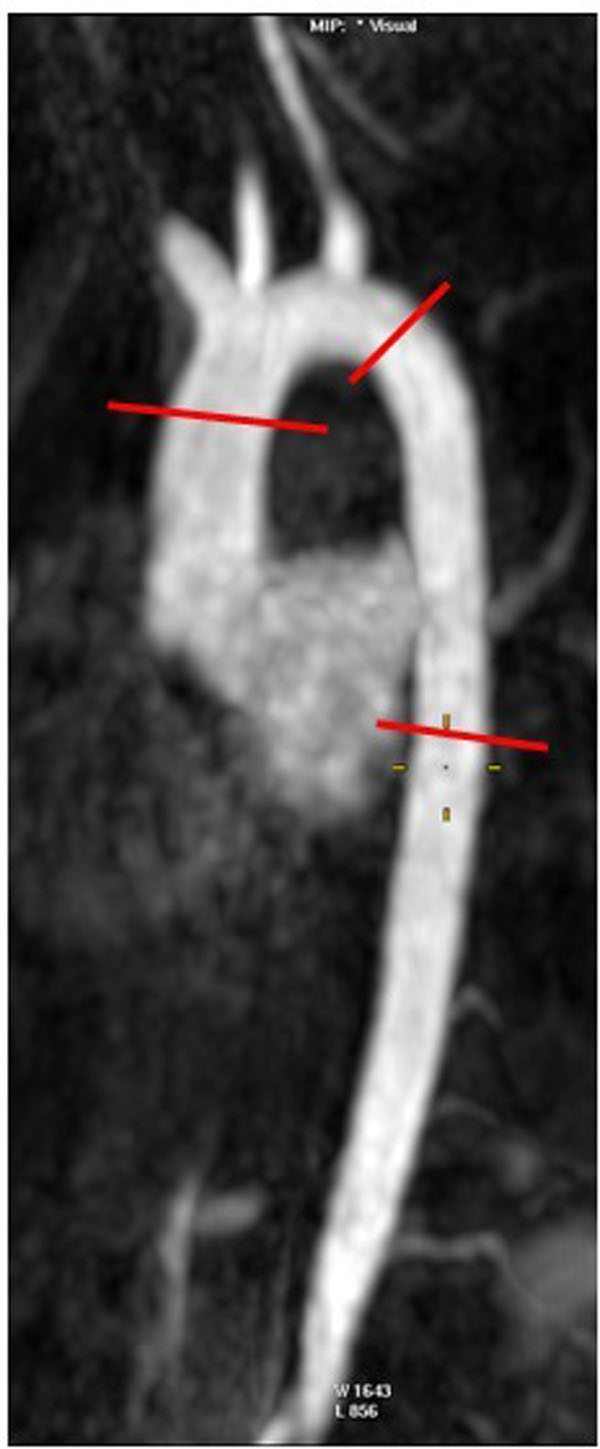


**Figure 2:**